# The elimination of apoptotic sperm in IVF procedures and its effect on pregnancy rate

**DOI:** 10.5935/1518-0557.20190007

**Published:** 2019

**Authors:** Martha Merino-Ruiz, Felipe Arturo Morales-Martínez, Edith Navar-Vizcarra, Otto H. Valdés-Martínez, Luis H. Sordia-Hernández, Donato Saldívar-Rodríguez, Oscar Vidal-Gutiérrez

**Affiliations:** 1 University Center for Reproductive Medicine, University Hospital "Dr. José Eleuterio González" of the UANL, Monterrey Nuevo León, México

**Keywords:** annexins V, MACS, ICSI

## Abstract

**Objective:**

To identify the effect of apoptotic sperm elimination with MACS in patients that require IVF.

**Methods:**

An experimental, cross-sectional, descriptive, prospective and non-blinded study of diagnostic tests performed in patients who required IVF and ICSI from July 2011 to July 2012. Ninety-two couples participated according to the treatment administered to the semen sample; in the control group: the samples were subjected only to density gradients before ICSI, in the study group: the same procedure was performed plus the addition of the MACS technique. Comparing the groups, we assessed the fertilization, division, viable embryos and clinical pregnancy rates in all cases.

**Results:**

We found significant differences when using MACS technique in sperm parameters. We found no differences between the total samples of the control and study groups. When separating the own and donated eggs in each group, we found an improvement in the fertilization rates (*p*<0.001) of the own eggs. In both groups, the handling of donated eggs lead to a significant improvement in the immunological pregnancy test (IPT) and fetal heart rate (FHR) results. Only in the donated eggs group, where MACS was applied, could we see that all cases with positive IPT had a fetal heart rate, which shows a significant difference (*p*<0.002) when compared with the control group, where the percentage decreased abruptly.

**Conclusions:**

This study demonstrates the effectiveness of the use of annexins (MACS) in eliminating apoptotic sperm, and when the obtained sperm is applied to good-quality eggs.

## INTRODUCTION

Advances in assisted reproduction techniques (ART) have been increasing in recent years; however, success rates have not yet improved. With the introduction of in vitro fertilization (IVF), especially with intracytoplasmic sperm injection (ICSI), the problem caused by the male factor is partially solved, but we are still far from offering a diagnostic and therapeutic effectiveness of 100% (Romany *et al*., 2009). In recent years, the sperm DNA nuclear integrity has been studied as a cause of male infertility. (Agarwal *et al*., 2003; Portella & Sepúlveda, 2011) In these cases, it is inferred that the problem is of molecular origin. (Said *et al*., 2008).

The currently applied methods of sperm selection bear controversial results, among which are: Physiological Intracytoplasmic Sperm Injection (PICSI), which uses the union of healthy sperm with hyaluronic acid (HA) and facilitates the selection of mature sperm. Different studies have reported that spermatozoids selected by binding to HA show less DNA fragmentation, less frequent chromosomal aneuploidies and good nuclear morphology (Parmegiani *et al*., 2010; Portella & Sepúlveda, 2011; Agarwal & Allamaneni, 2005); intra-cytoplasmic morphologically-selected sperm injection (IMSI). The method used for detailed morphological evaluation of real-time mobile sperm is called MSOME (Motile sperm organelle morphology examination), performed with a microscope with a 6300x magnification. The normality of the sperm nucleus reflects its DNA content and organization, which may influence the results of IVF/ICSI procedures (Bartoov *et al*., 2003; Romany *et al*., 2009; Figueira *et al*., 2011) and the selection of magnetically activated cells (MACS) by the use of annexin V3 as a marker of early apoptosis in mature sperm from infertile patients. (Agarwal *et al.*, 2003; Said *et al*., 2008).

Recent clinical studies (de Vantéry Arrighi *et al*., 2009; Grunewald *et al*., 2006) indicate levels of DNA fragmentation above 30%, as measured by a sperm chromatin structure assay (SCSA), decreasing the possibility of initiating and maintaining the course of a full-term pregnancy (Chirinos *et al*., 2007); therefore, it is considered necessary to select non-fragmented sperm in ICSI. The MACS technique is a non-invasive method for separating sperm cells that contain fragmented DNA because of an apoptotic process. It can be useful in couples with male infertility, poor embryo quality in previous cycles of IVF or in cases of unexplained infertility. (Agarwal & Allamaneni, 2005; Miltenyi *et al*., 1990; Said *et al*., 2005; 2006).

A different approach has been applied to the selection of activated cells (MACS) when using it as a preparation technique that selects mobile, morphologically normal, viable spermatozoa that show higher rates of survival to cryopreservation, as well as greater potential of fertilization. (Agarwal *et al*., 2003).

Our main objective is to identify the effects of eliminating the apoptotic sperm after applying the MACS technique to the semen of couples with infertility that require IVF-ICSI and observe the results in the fertilization rates, embryo quality, immunological pregnancy test (IPT) and clinical pregnancy.

## MATERIAL AND METHODS

### Design

An experimental, cross-sectional, descriptive, prospective, non-blinded, diagnostic test was conducted in infertile couples who attended the University Center for Reproductive Medicine, University Hospital, U.A.N.L. requiring IVF-ICSI, from July 2011 through July 2012.

### Patients

95 couples from unselected males signed an informed consent form and the Ethics Committee of the Faculty of Medicine and University Hospital "Dr. José Eleuterio González" under the number GI11-015 approved the study.

### Procedure

The pairs were grouped according to the treatment administered to the semen sample. In the control group, the samples were submitted only to density gradients before the ICSI, while in the study group, the same procedure was performed plus the application of the MACS technique according to commercial house instructions (MACS Dead Cell Removal Kit, Miltenyi Biotec Inc.). In the study group, motility, vitality and morphology were evaluated using the Papanicolaou technique before and after the MACS procedure. The evaluation was carried out according to the WHO criteria (Cooper *et al*., 2010). In order to identify some male factor pathology in the study group, a survey was applied on important previous history that could affect or cause infertility.

In order to perform IVF-ICSI in all cases, the couples underwent COS (controlled ovarian stimulation) according to the protocol established in our center. The eggs obtained were retrieved in buffered media GlobalW/Hepes (Life global, USA) supplemented with 5% of Human serum albumin (HAS) and transferred to a Global total for Fertilization (Life global, USA) and were sub classified according to the source: donor or own eggs; mature oocytes were injected with sperm according to the standard ICSI protocol of our center. In the latter, the woman's age was considered. Fertilization was checked at 16-18 h post insemination, checking the presence of 2PN; the embryos were then prolonged in the culture medium covered with mineral oil (Life global, USA) from days 1 to 3. Embryo transfer was performed on day 3 and extra embryos were vitrified on day 3. The fertilization rate, cell division rate, viable embryos, IPT, and clinical pregnancy with FHB on ultrasound 2 to 3 weeks after positive BhCG were evaluated in all cases.

### Statistical analysis

The results obtained were plotted in a database developed in Microsoft Excel 2010 software, for further analysis using the IBM SPSS Statistics software version 20. The frequencies and descriptions of the continuous categorical and numerical variables were analyzed. The tests of statistical significance were performed for categorical variables using the chi-square. The continuous numerical variables were analyzed by the Student's T test for related samples. The study values were segmented and contrasted according to the administration of annexins through hypothesis tests for means and proportions, depending on the case for each type of variable (quantitative and qualitative, respectively) at a reliability rate of 95%. The multivariate association was evaluated by linear logistic regression at the same reliability rate.

## RESULTS

The initial analysis included 95 cases, of which 3 were excluded - 2 because of lack of eggs recovered and 1 in which embryo transfer was not possible due to ovarian hyperstimulation syndrome. A total of 92 cases were analyzed, 46 in the control group and 46 in the study group. In relation to the questionnaire that was applied to the males of the study group, we found that only seven cases had any identifiable pathology: two with a history of varicocele, two with metabolic disorders (diabetes), one with exposure to cocaine and tobacco and two with functional testicular problems. Table 1 shows the comparative analysis of the variables in both study groups. No significant difference was detected.

**Table 1:** Description of the variables in the study and control group

VARIABLE	Study group average (range)	Control group average (range)	*p*
n	46	46	
Women age (years)	36 (24-46)	36 (20-47)	NS
Male age (years)	37 (24-53)	39.7 (30-58)	NS
Total ovules	8.5 (1-31)	9.7 (3-28)	NS
Oocytes MII	6.3 (0-22)	6.8 (1-18)	NS
Fertilization	5.0 (1-10)	4.5 (1-10)	NS
Division	4.2 (1-9)	3.9 (1-9)	NS
Transferred embryos	2.07 (1-3)	2.3 (1-4)	NS
Vitrified embryos	1.0 (0-4)	1.0 (0-7)	NS
Viable embryos	3.1 (1-6)	3.2 (1-9)	NS
Counts/10^6^per ml (in fresh)	128 (1-470)	78 (0.5-248)	NS
Mobile forms /10^6^per ml (fresh)	121 (18-486)	72 (1-488)	NS
Counts/10^6^per ml (post capacitation)	52 (7-216)	30 (0.4-123)	NS
Mobile forms/10^6^ per ml (post capacitation)	43 (0.1-100)	35.4 (0.3-100)	NS

**Figure 1:**
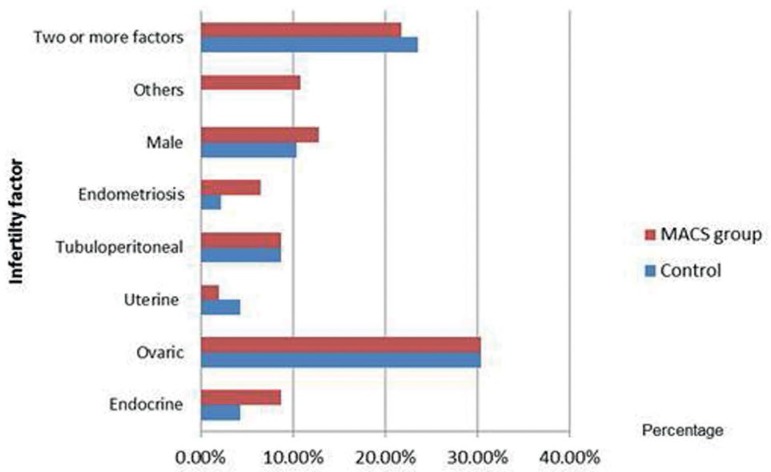
Infertility couple factors

The etiology of couple infertility was analyzed by subdividing the groups according to their infertility factor. The main factors identified where the ovarian in both groups, followed by the male and the combination of two or more factors (Figure 1).

Semen was compared before and after the annexin technique to assess motility, vitality and morphology using the Papanicolaou stain. There was quality improvement (*p*=0.001) in all cases when using the MACS technique (Table 2).

**Table 2:** Analysis of semen samples in fresh and subsequent to MACS technique in relation to motility, vitality, and morphology

	Mean	Standard deviation	Student's T	*p*
MOT-F/MOT-A	-11.26087	20.76903	-3.677	0.001*
VIT-F/VIT-A	-3.41304	10.67203	-2.169	0.035*
PAP-F/PAP-A	-7.11364	7.74361	-6.094	0.000*

* With statistical significance (*p*<0.05)

MOT-F / MOT-A: fresh motility / motility with annexins. VIT-F / VIT-A: fresh vitality / vitality after annexins. PAP-A: staining with Papanicolaou technique in fresh / staining with Papanicolaou after annexin.

When comparing the total cases of both groups and evaluating the percentage of fertilization, division and number of embryos, no significant difference was found between the groups, only a tendency to improve the percentage of fertilization in the study group, we need more patients to see differences that could change the percentage.

However, by separating the groups according to their own or donated eggs, the percentage of fertilization in the group with own eggs improved significantly. It is interesting that with donated eggs the percentage of viable embryos without annexins is higher (Table 3).

**Table 3:** Analysis of the study groups subdivided into own versus donated eggs in relation to the rate of fertilization, division and viable embryos

	OWN			DONATED		
	Control group	Study group	*p*	Control group	Study group	*p*
% fertilization	67	80	0.001*	75	79	0.502
% division	86	82	0.440	87	80	0.182
% viable embryo	83	74	0.121	81	69	0.050*

* *p*<0.05 with statistical significance.

When comparing the results of the immunological pregnancy test (IPT+) between both groups, correlated with the presence of a clinical pregnancy with fetal heart rate (FHR+), no significant difference was found.

The results were compared with the MACS technique and its application between own and donated eggs. Table 4 shows that in both cases, the handling of donated eggs leads to a significant difference in the results when evaluating IPT and fetal heart rate.

**Table 4:** Analysis of the pregnancy course with own vs donated eggs

	Own eggs	Donated eggs	*p*
Control group			
IPT (+)	6 (20.7%)	9 (52.9%)	0.015*
FHR (+)	5 (17.2%)	5 (29.4%)	0.014*
Study group			
IPT (+)	7 (23.3%)	8 (50.0%)	0.0016*
FHR (+)	5 (20.6%)	8 (50.0%)	0.0002*

* Statistical significance (*p*<0.05)

Using MACS, Table 5 shows that only in the donated egg group, when evaluating IPT (+) associated with the presence of FHR (+), the pregnancy course is significantly different when compared to the control group, in which the percentage of clinical pregnancy decreases abruptly.

**Table 5:** Results of the IPT in the groups of patients with donated eggs

	Study group n=46	Control group n=46	*p*
Total cases IPT (+)	15 (32.6%)	15 (32.6%)	1
Donated eggs IPT (+)	8 (50%)	9 (52.9%)	0.67
Donated eggs FHR (+)	8 (50%)	5 (29.4%)	0.002*

* Statistical significance (*p*<0.05)

## DISCUSSION

The MACS technique selects spermatozoa with deteriorated membranes and with externalization of phosphatidylserine as a manifestation of apoptosis acting at the molecular level and complementing the sperm preparation protocol in ART. There are very few studies that evaluate the effectiveness of sperm separation with apoptosis using the MACS technique in ICSI cycles in patients with their own and donated eggs, hence the importance of the present study.

Said *et al*. (2005) evaluated the effects of MACS in samples of healthy voluntary donors before and after cryopreservation, finding in the fresh annexin-negative sample an improvement with statistical significance in relation to motility (*p*<0.006), and survival rate after of cryopreservation (*p*<0.04) when compared to the annexin-positive group only when MACS was applied prior to cryopreservation, since applying it after, caused an important decrease in the motility percentage (Grunewald *et al*., 2009). Our results are similar in relation to sperm motility in the annexin-negative group. The samples were analyzed in infertile men, and we found that by eliminating the damaged or apoptotic sperm, motility improved.

Said *et al*. (2006) evaluated the fertilization potential with spermatozoa treated with annexin V negative (not apoptotic) to determine if the use of MACS before the ART procedures improved the success rates. The degree of DNA fragmentation (caspase 3 levels, MMP integrity and DNA fragmentation) was measured in the groups: positive annexins, negative annexins and control group. The degree of spermatozoa penetration in the different groups in hamster oocytes was tested freely and by ICSI. The results in sperm motility values were significantly higher in annexin V-negative (*p*<0.001) patients, as well as among controls (*p*<0.007). A higher percentage was found in this group of fertilized oocytes *p*<0.001 (Said *et al*., 2005). On the other hand, de Vantéry Arrighi *et al*. (2009) analyzed the semen samples of infertile patients and found 70% spermatozoa reduction with externalization of the phosphatidylserine in their membrane, with a 50% increase in post-selection survival. The samples subjected to annexins in our study improved in motility, vitality and morphology.

Álvarez Sedó et al. (2011) conducted a study in males with positive apoptosis markers, in order to assess whether MACS effects are impacted by oocyte quality depending on age (group 1 <37 years and group 2 > 37 years). They found a significant difference between both groups with improvement in embryo quality, pregnancy and implantation rates in group 1. In our study, we found the same effect in the two treated groups, the highest number of pregnancies was achieved in women under 37 and only one case in a 39-year-old woman.

Arnanz *et al*. (2012) studied patients with own (22 patients) and donated eggs (11 patients) in infertile couples with ICSI after MACS, in which they considered fertilization and not pregnancy rates, showing that the number of fertilized oocytes were increased with the use of MACS before ICSI only in the own eggs group. In our study, we also found an improvement in the fertilization rate of the own-eggs group, receiving sperm treated with annexins. Our results also show no difference in fertilization rates, although they do show a significant increase in the achievement of pregnancies in the cases of donated eggs treated with annexins.

Dirican *et al*. (2008) studied 196 infertile couples with oligoasthenozoospermia to which annexins were used before ICSI, and to whom assisted, hatching was also performed. Subsequently, a significant improvement was found in the sperm morphology and the division rate, with a non-significant improvement in clinical pregnancy. In the total cases of our study, we found a significant improvement in the fertilization rate, but not in the division or pregnancy rates. We did not use assisted hatching (technique that helps improve embryo implantation); however, a significant difference was found in the clinical pregnancy rate in cases of patients with donated eggs.

## CONCLUSIONS

Significant differences were found in the semen samples before and after using annexins in the vitality, motility and morphology rates. The fertilization rate improved in the cases treated with annexins and own eggs, but there was no improvement in the division rate and embryo quality. In the clinical pregnancy rate, a significant improvement was found only in the cases of donated eggs and spermatozoa treated with annexin V. This study demonstrated that the effectiveness of annexins in eliminating apoptotic sperm is manifested when treated sperm is applied to good quality eggs. These results are encouraging and suggest that the application of this technique can increase the number of clinical pregnancies. More studies with a greater number of cases should be done to confirm these results.
